# The role of secure instant messaging applications in medical education: Evaluating student satisfaction in a case-based learning program using Siilo

**DOI:** 10.3389/fmed.2023.1139859

**Published:** 2023-03-01

**Authors:** Tali Shahar, Offer Tadmor, Uri Dior, Shay Porat, Myriam Safrai, Yosef Ezra, Doron Kabiri

**Affiliations:** ^1^Department of Obstetrics and Gynecology, Hadassah-Hebrew University Medical Center, Jerusalem, Israel; ^2^Maccabi Health Services, Jerusalem, Israel

**Keywords:** medical education, instant messaging, mobile application, case-based learning, Siilo application

## Abstract

**Background:**

Instant messaging applications for mobile phones have recently grown in popularity among medical personnel, including both physicians and medical students. During the COVID-19 pandemic, medical education was largely transferred to virtual platforms, making such applications an increasingly important tool for medical education. “Siilo” is a secure instant messaging application that was designed for medical professionals, and offers several advantages over other instant messaging services that are vital for its use in medical settings, including information security, data encryption, and a built-in blurring tool to maintain patient privacy. In addition, Siilo allows for the creation of individual folders for each case, enabling users to conduct separate discussions about multiple patients simultaneously.

**Objective:**

To evaluate student satisfaction in a case-based learning program using Siilo as a medical education tool in improving student learning outcomes and motivation.

**Methods:**

A case-based learning program was conducted with 24 fifth-year medical students using Siilo to evaluate its effectiveness as a medical education tool. The program was evaluated through the use of pre- and post-program questionnaires and focus group discussions to assess student satisfaction.

**Results:**

The majority of students (83.3%) were highly satisfied with the Siilo platform and felt that it enhanced their learning experience, and a majority of students (79.1%) reported that the program was highly effective. Students reported that the platform was easy to use and provided a clear and organized way to follow discussions about cases. The focus group discussions further revealed that students appreciated the real-time communication and felt that the use of Siilo helped to improve the quality of communication and collaboration during the learning process. The use of Siilo as a medical education tool was found to contribute to positive relationships between doctors and students and improve student motivation for learning and outcomes.

**Conclusion:**

These findings suggest that Siilo can be a valuable resource for medical education, particularly due to its secure and convenient features, which are well-suited for use in medical settings. The use of Siilo in a case-based learning program was found to be effective in improving student satisfaction and learning outcomes and contributed to positive relationships between doctors and students. These results highlight the potential for utilizing mobile instant messaging apps as a tool for enhancing clinical teaching in medical education.

## Introduction

Instant messaging apps for mobile phones have gained popularity among medical professionals in recent years, particularly during the COVID-19 pandemic when medical education was largely conducted virtually (1). “Siilo” is a secure instant messaging app specifically designed for medical professionals, with features such as data encryption and personal password protection to ensure medical confidentiality. The app’s interface is partially modeled after the popular instant messaging application WhatsApp (2).

Siilo has several features that make it an optimal choice for use in the medical field. Information security and data encryption are given high priority to ensure medical confidentiality. The application meets European standards for information security and requires a personal password for each login (3). User authentication is conducted through verification of medical license numbers and additional personal details. In addition, Siilo includes a built-in blurring tool to maintain patient privacy by obscuring personal details. These features demonstrate Siilo’s commitment to security and confidentiality in medical communication.

One of Siilo’s particularly useful features for medical education is the ability to create individual folders for each case. These folders can store text messages and relevant media files, such as pictures, videos, and documents, allowing users to easily follow discussions about each patient. This feature enables users to conduct separate discussions about multiple patients at the same time, without the risk of confusion. In summary, this function enhances the organization and clarity of medical discussions on the platform.

Siilo offers convenience, as cases can be accessed at any time and location, and discussions can be conducted asynchronously. This allows for the possibility of student groups being gathered from multiple sites within or outside their country. Additionally, Siilo is compatible with any smart mobile device and a desktop version has been developed as well, making it widely accessible. These features contribute to the user-friendliness of the platform.

Several studies have examined the use of instant messaging applications in medical education and have reported increased motivation for learning (4, 5), higher satisfaction with the learning process (6–8), and improved outcomes (e.g., better test scores) (5, 8) among students who used such tools. These applications have also been shown to foster positive relationships between doctors and students (9).

Given the prevalence of digital communication in the lives of students and physicians, and the unique advantages of Siilo over other instant messaging applications, we sought to examine how its special features could be utilized to enhance clinical teaching of medical students through a case-based learning program developed on the Siilo platform. Our primary objective was to evaluate student satisfaction with this approach.

## Methods

This was a survey-based study, including details of the survey questionnaire. A total of 24 fifth-year medical students participated in the case-based learning program using Siilo.

A Siilo group was created for fifth-year medical students who were completing their clinical rotation in the Obstetrics and Gynecology Department. After acquiring the necessary theoretical knowledge, these students were invited to participate in case-based educational activities during the last week of their clinical rotation. These activities included three evolving cases that were specifically prepared for the students on the topics of gynecologic ultrasounds, obstetrics, and fertility. Each case was introduced with a brief description and related questions, and it evolved based on students’ questions and additional relevant data.

To measure the students’ engagement and activity in the case-based learning, we collected data on the number of students actively or passively participating in the case-based learning, the number of messages exchanged, the number of media files shared, and the average time spent on the Siilo platform. We also tracked the evolution of the cases by monitoring the progression of the discussions and the students’ questions, in order to assess the effectiveness of the case-based learning approach in promoting critical thinking and problem-solving skills.

### Case details

Three cases were included in the case-based educational activities, covering the topics of gynecologic ultrasound, obstetrics, and fertility.

The gynecologic ultrasound case consisted of 54 messages, including 37 text messages, seven ultrasound videos, six ultrasound images, two graphs, one PDF file, and one link to a YouTube video. One physician and seven students actively participated in this case, while seven additional students observed without active participation.

The obstetrics case included 78 messages, comprising 68 text messages, three ultrasound images, two PDF files, two graphs, two diagrams, and one picture. Nine students participated in the discussion, and five additional students observed without active participation.

The fertility case contained 108 messages, including 107 text messages and one photo. Nine students participated in the discussion, and five observed without active participation.

### Data collection

The data collection for this study involved administering a questionnaire consisting of four questions rated on a five-point Likert scale. The purpose of the questionnaire was to assess the effectiveness of the case-based learning approach in promoting critical thinking and problem-solving skills among the participants. The questionnaire consisted of two questions prior to the training, aimed at evaluating participants’ expectations, and post-training, four questions, administered to assess the effectiveness of the case-based learning approach in promoting critical thinking and problem-solving skills.

The pre-training questions were as follows: (1) “I anticipate that participating in the case-based learning program during the round will enhance my learning experience” and (2) “I believe that utilizing the Siilo instant messaging software will facilitate communication during the case-based learning program.” The post-training questions were as follows: (1) “Did the case-based practice with Siilo prove to be effective for you?” (2) “Did the case-based practice with Siilo adequately prepare you for the exam that took place at the end of the round?” (3) “Do you find the use of the Silo software to be convenient?,” and (4) “Would you be receptive to engaging in more practices through the Silo platform during the round?”

The study utilized a five-point Likert scale, with scores ranging from 1 to 5, to assess various aspects of the case-based learning program and the use of Siilo as an educational tool. The Likert scale was adapted to various questions, including efficacy, level of preparation, ease of use, and desire for additional training. The scores were interpreted as follows: Efficacy—1: Ineffective, 2: Somewhat Ineffective, 3: Effective, 4: Highly Effective, and 5: Extremely Effective; Level of Preparation—1: Not Prepared, 2: Poorly Prepared, 3: Adequately Prepared, 4: Well Prepared, and 5: Extremely Well Prepared; Ease of Use—1: Difficult to Use, 2: Challenging to Use, 3: Easy to Use, 4: Very Easy to Use, and 5: Extremely Easy to Use; Desire for Additional Training—1: No Desire, 2: Little Desire, 3: Some Desire, 4: Strong Desire, and 5: Extreme Desire. It is important to note that these scores were used consistently across all questions to ensure the comparability of results.

### Data analysis

The data analysis of the data collected from the pre- and post-program questionnaires was performed using an Excel sheet. To assess the effectiveness of this teaching method, we administered two surveys: one before each teaching session to evaluate participants’ expectations, and another after the session to gauge their satisfaction with the program. The data collected from the pre- and post-surveys were analyzed using a Likert scale as presented at the data collection section. Descriptive statistics, including statistical indices (means, percentages), were used to summarize the data. In addition, we conducted a qualitative analysis of the students’ questions and discussions on the Siilo platform, to evaluate the effectiveness of the case-based learning approach in promoting critical thinking and problem-solving skills. The focus group discussions were transcribed and analyzed using thematic analysis. The transcripts were read several times to identify recurring themes and patterns in the data. These themes were then organized to provide a comprehensive summary of the students’ perceptions and experiences.

## Results

A total of 24 fifth-year medical students participated in the case-based learning program using Siilo.

### Participants’ feedback

**Figure 1:**
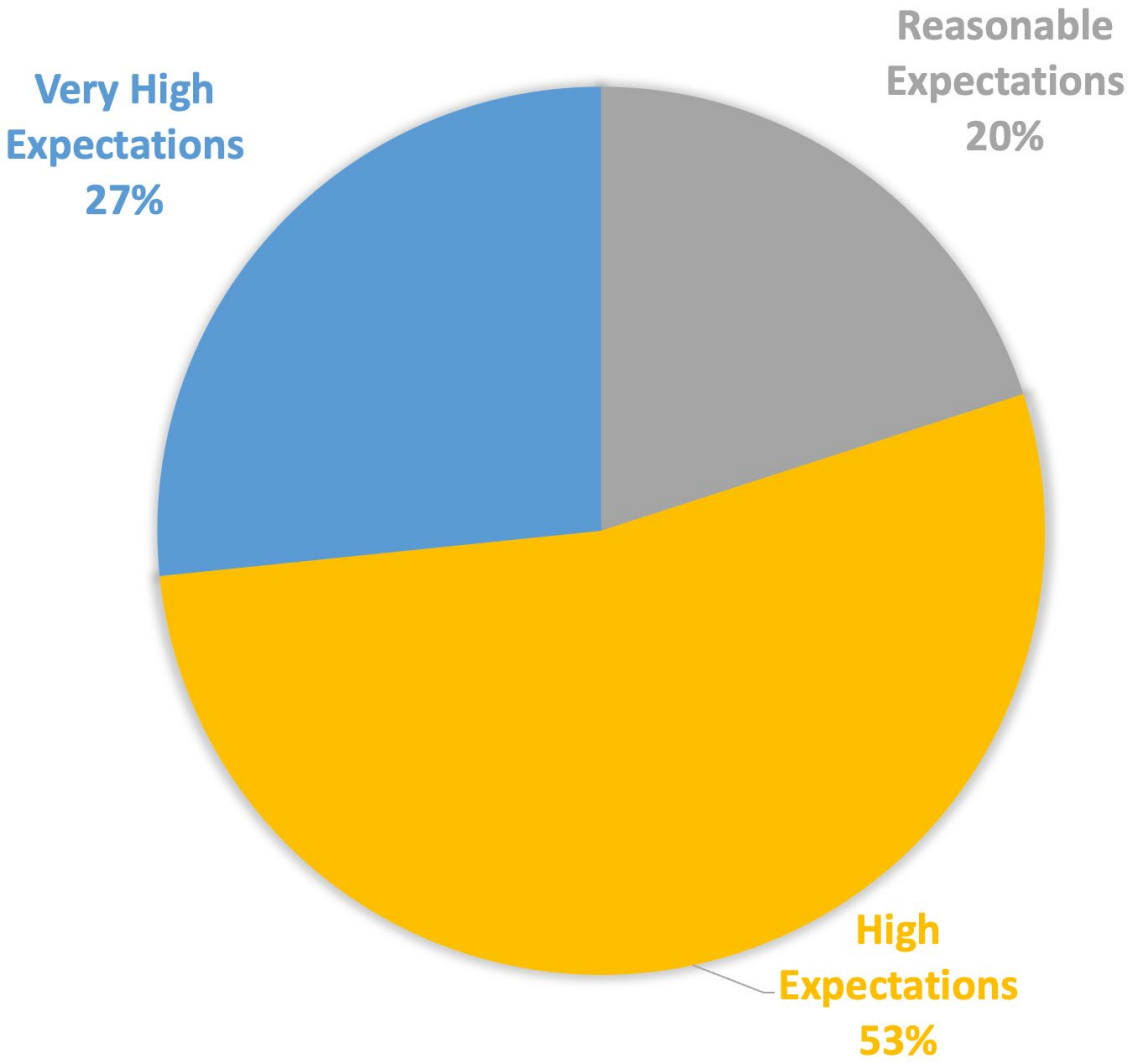
Students’ expectations before joining the case-based learning program. A survey was conducted among students before they joined the study, asking them to share their expectations. The graph shows that 80.0% of the respondents anticipated that participation in the study group would be beneficial.

Before joining the study groups, a majority of students (73.3%) believed that participating in evolving clinical cases during their ward rotation would aid in acquiring the required theoretical knowledge (Figure 1). Students expressed their expectations to “have a clear review of basic, common cases,” “practice clinical thinking and understand the implications of treatment options,” and “receive good clinical instruction with timely and helpful technical support, minimizing technical difficulties as much as possible.”

**Figure 2:**
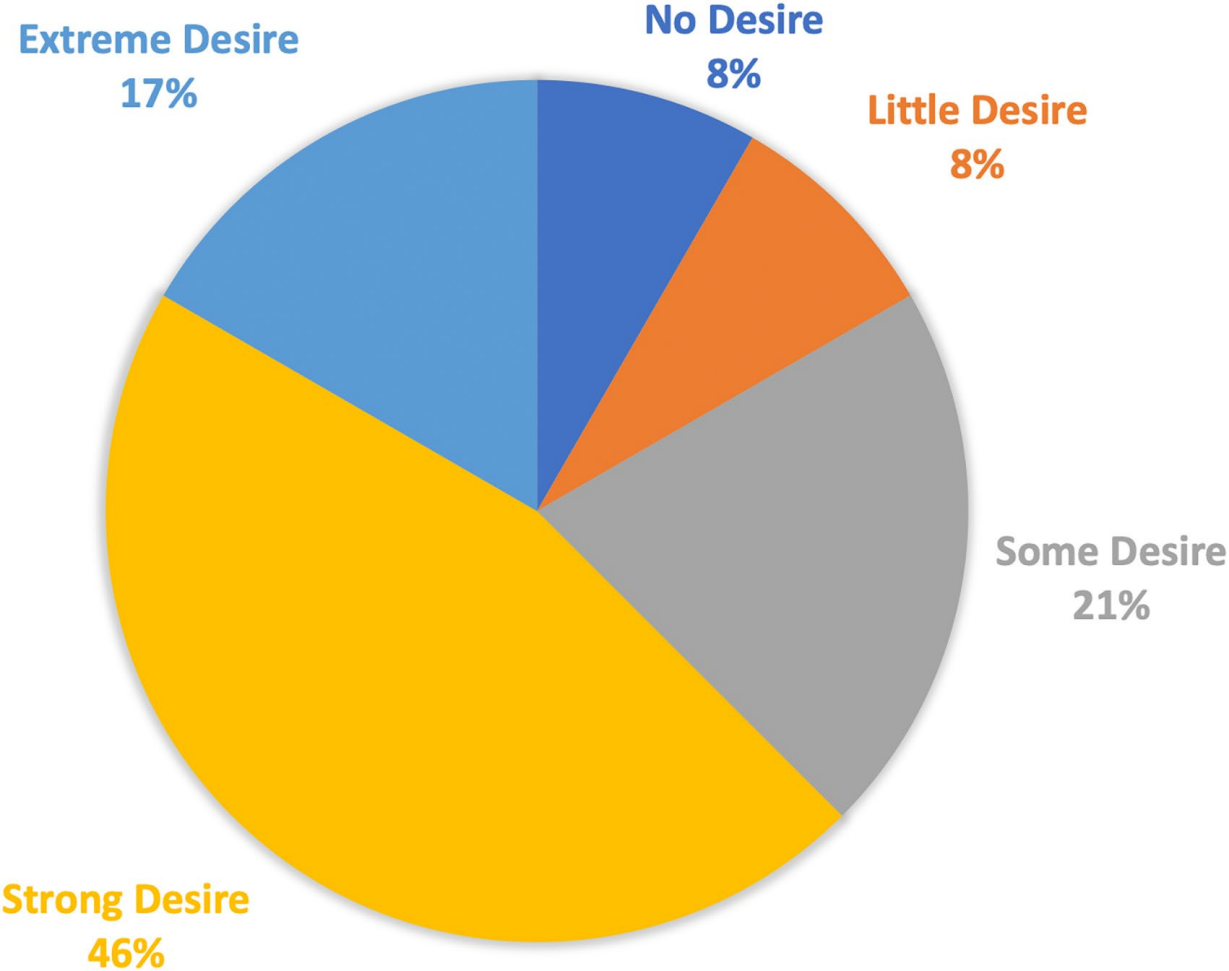
Students’ satisfaction after the study. A survey was conducted among students to assess their satisfaction with their participation in the study. The graph shows the percentage of students who expressed a desire to participate in another session.

Following the completion of the learning sessions, a majority of students (79.1%) reported that the program was effective, highly effective, or extremely effective, with a mean satisfaction score of 3.4 on a Likert scale. The majority of students (83.3%) believed that the “Siilo” case studies contributed to their exam preparedness, with a mean of 3.41 on a Likert scale. In addition, 87.5% found “Siilo” easy, very easy, or extremely easy to use, with a mean of 3.66 on a Likert scale. A large proportion of students (80.0%) expressed some, strong, or extreme desire to participate in additional “Siilo” case studies during their clinical rotations, with a mean of 3.54 on a Likert scale (Figure 2).

When asked to summarize their overall experience, several students provided insightful feedback on the case-based learning program and the Siilo instant messaging software. One student stated, “This platform is excellent for learning and practicing during clinical rotations.” Another student praised the design of the exercise, saying, “The exercise was well done and covered a range of important topics.” A third student commented, “I believe the program was excellent and would have benefited from additional opportunities.” These comments provide valuable insights into the effectiveness and value of the case-based learning approach and the Siilo software in promoting critical thinking and problem-solving skills among medical students.

## Discussion

In this study, medical students reported a high level of satisfaction with case-based learning using the “Siilo” instant messaging mobile application. Students’ participation, cooperation, and self-reported satisfaction on a feedback questionnaire demonstrated the effectiveness of the experimental educational program. The majority of students found the teaching sessions easy to use and believed they contributed to their exam success. There was a strong desire among students to participate in similar educational exercises in the future.

Multiple studies have explored the use of communication applications in medical education. Zulfikar et al. (4) found that the use of instant messaging mobile applications increased motivation and knowledge among students. Dar et al. (6), Meerasai and Mohesh (7) and Hossain et al. (9) all demonstrated increased student satisfaction with learning using instant messaging applications and improved relationships between medical students and doctors. Mohanakrishnan et al. (10) found messaging applications to be effective for transferring relevant information and preparing for frontal lectures, and Dyavarishetty and Patil (5) observed higher exam scores among students who participated in case-based learning through educational messaging groups compared to their non-participating peers.

This study represents the first evaluation of the use of the “Siilo” instant messaging application for virtual case-based teaching sessions for medical students. The “Siilo” app, specifically designed for use by medical professionals, was found to be a user-friendly, secure platform suitable for teaching clinical skills to students. It allows users to create and manage separate case discussions in parallel, and its use may help to educate future doctors on the importance of secure communication tools for protecting patient privacy. Additionally, the “Siilo” app has the potential to facilitate the organization and management of complex medical cases that require collaboration among multiple teams, through the use of a dedicated, orderly platform.

This study has several limitations that should be considered when interpreting the results. First, the sample size of the study is small, which could affect the generalizability of the findings. Second, internet access is required to use the “Siilo” application, which may be limited in certain areas. Additionally, the small screen size of smartphones may make it difficult to view images with fine details or figures with many items. Finally, not all students may have smartphones that are suitable for using the “Siilo” app, although a desktop version of the app is available.

To further assess the effectiveness of “Siilo” as an educational tool, future research should consider evaluating the application’s satisfaction levels among a larger sample of students. Additionally, it would be valuable to compare the success rates of students who utilized “Siilo” in their education with those who did not. It would also be useful to examine the efficiency and ease of use of the application among medical teams managing real-time cases.

## Conclusion

The “Siilo” instant messaging application appears to be a promising tool for facilitating case-based learning in a medical setting. The application, designed specifically for use by medical professionals, was found to be user-friendly and secure, with a high level of satisfaction reported by participants. These findings suggest that the use of “Siilo” in medical education may be an effective way to enhance clinical instruction for students. Future research should explore the use of “Siilo” in larger student samples and compare its effectiveness to other methods of medical education. In addition, further studies should evaluate the efficiency and ease of use of “Siilo” among medical teams providing real-time medical case management.

## Data availability statement

The raw data supporting the conclusions of this article will be made available by the authors, without undue reservation.

## Ethics statement

The studies involving human participants were reviewed and approved by Hadassah IRB. Written informed consent for participation was not required for this study in accordance with the national legislation and the institutional requirements.

## Author contributions

TS, OT, UD, SP, MS, YE, and DK reviewed the literature and wrote the paper. DK performed the statistical analyses for this study. TS and DK designed the data collection. All authors contributed to the article and approved the submitted version.

## Conflict of interest

The authors declare that the research was conducted in the absence of any commercial or financial relationships that could be construed as a potential conflict of interest.

## Publisher’s note

All claims expressed in this article are solely those of the authors and do not necessarily represent those of their affiliated organizations, or those of the publisher, the editors and the reviewers. Any product that may be evaluated in this article, or claim that may be made by its manufacturer, is not guaranteed or endorsed by the publisher.

## Abbreviations

COVID-19: Coronavirus disease 2019
